# Amino-functionalized vertically ordered mesoporous silica film on electrochemically polarized screen-printed carbon electrodes for the construction of gated electrochemical aptasensors and sensitive detection of carcinoembryonic antigens

**DOI:** 10.3389/fchem.2024.1490940

**Published:** 2024-11-11

**Authors:** Ke He, Hongxin Wang, Tao Luo, Fei Yan, Jing Guo

**Affiliations:** ^1^ Guangxi Medical University Cancer Hospital, Nanning, China; ^2^ Department of Chemistry, School of Chemistry and Chemical Engineering, Zhejiang Sci-Tech University, Hangzhou, China; ^3^ Department of Dermatology, Shandong Provincial Hospital Affiliated to Shandong First Medical University, Jinan, Shandong, China

**Keywords:** amino-functionalized vertically ordered mesoporous silica film, screen-printed carbon electrode, electrochemical polarization, carcinoembryonic antigen, electrochemical aptasensor

## Abstract

Disposable electrochemical biosensors with high sensitivity are very fit for point-of-care testing in clinical diagnosis. Herein, amino-functionalized, vertically ordered mesoporous silica films (NH_2_-VMSF) attached to an electrochemically polarized screen-printed carbon electrode (p-SPCE) are prepared using a simple electrochemical method and then utilized to construct a gated electrochemical aptasensor for rapid and sensitive determination of carcinoembryonic antigen (CEA). After being treated with the electrochemical polarization procedure, p-SPCE has plentiful oxygen-containing groups and improved catalytic ability, which help promote the stability of NH_2_-VMSF on SPCE without the use of an adhesive layer and simultaneously generate a highly electroactive sensing interface. Owing to the numerous uniform and ultrasmall nanopores of NH_2_-VMSF, CEA-specific aptamer anchored on the external surface of NH_2_-VMSF/p-SPCE serves as the gatekeeper, allowing the specific recognition and binding of CEA and eventually impeding the ingress of electrochemical probes [Fe(CN)_6_^3−/4−^] through the silica nanochannels. The declined electrochemical responses of Fe(CN)_6_^3−/4−^ can be used to quantitatively detect CEA, yielding a wide detection range (100 fg/mL to 100 ng/mL) and a low limit of detection (24 fg/mL). Moreover, the proposed NH_2_-VMSF/p-SPCE-based electrochemical aptasensor can be applied to detect the amount of CEA in spiked human serum samples, which extends the biological application of a disposable NH_2_-VMSF/p-SPCE sensor by modulating the biological recognition species.

## 1 Introduction

Vertically ordered mesoporous silica films (VMSF), called silica isoporous membranes, consist of many regularly and perpendicularly aligned nanochannels parallel to each other (Walcarius, 2021; Zhou et al., 2020). These nanochannels are ultrashort (∼100 nm), and their diameter is ultrasmall (2–11.8 nm), making VMSF a promising electrode modification material in the field of electrochemical analysis (Fan et al., 2024; Duan et al., 2024; Wei et al., 2022; Zhang et al., 2023a). Benefiting from the permselectivity at the molecular level, VMSF not only greatly increases the amounts of small electroactive molecules near the electrode via electrostatic (Huang et al., 2024a; Luo et al., 2022; Yu et al., 2024), lipophilic (Sun et al., 2016), or hydrogen bond (Zheng et al., 2022) effects but also exhibits excellent anti-biofouling capacity in biological samples without complicated pretreatment procedures. VMSF, as a rigid structure, has two independent regions, namely, tiny internal nanochannels and the external surface. The uniform nanospace afforded by silica nanochannels can accommodate nanostructures [e.g., graphene quantum dots (Zhang et al., 2023b), metal nanoparticles (Zhou et al., 2024; Zhang et al., 2023c; Chang et al., 2023; Zhang et al., 2024), and conductive polymers (Ding et al., 2014)] for enhanced analytical performance and simultaneously permit the access of electrochemical probes/electrochemiluminescence luminophores for signal generation. The external surface of VMSF can be modified with biological species [e.g., enzymes (Huang et al., 2024b; Ma et al., 2024), antibodies (Chen et al., 2023; Xing et al., 2024; Huang et al., 2023a), antigens (Gong et al., 2022), and aptamers (Li et al., 2023; Zhang et al., 2023d; Xing et al., 2023; Zhou et al., 2023; Ma et al., 2023)] through covalent or electrostatic effects to form a target-specific interface, and the signal variation of electrochemical probes/electrochemiluminescence luminophores can be introduced. The above characteristics endow VMSF with a unique potential for developing various electroanalytical strategies.

In general, Stöber-solution growth and electrochemically assisted self-assembly (EASA) methods are two common bottom-up approaches for fabricating VMSF on the electrode surface (Teng et al., 2012; Walcarius et al., 2007). The former method requires the substrate electrodes carrying negative charges [e.g., indium tin oxide (ITO) and glass], and the latter one needs conductive electrodes [e.g., ITO (Ma et al., 2022a; Zeng et al., 2023), gold (Yan et al., 2022), glassy carbon electrodes (Huang et al., 2023b), or screen-printed carbon electrodes (SPCEs) (Ma et al., 2022b)]. As for the instability issue of VMSF on the carbonaceous electrodes, the introduction of oxygen-containing moieties on the electrode surface, including pre-activation procedures (Su et al., 2022; Zhu et al., 2022; Deng et al., 2023) or adhesive layers [silane molecules (Nasir et al., 2016), two-dimensional graphene nanosheets (Lv et al., 2022; Ma et al., 2022c) and their nanocomposites (Zhou et al., 2022)] have been employed. As reported previously, the electrochemical polarization of SPCE is helpful for the stable growth of VMSF, and the obtained VMSF/p-SPCE has been selected as sensitive anti-biofouling sensors for the detection of small electroactive species (Wang et al., 2022). To the best of our knowledge, such VMSF/p-SPCE has not been designed for gated electrochemical biosensors.

In this work, we propose the use of VMSF-bearing amino groups on the p-SPCE (NH_2_-VMSF/p-SPCE) for the construction of a disposable gated electrochemical aptasensor. p-SPCE offers the electroactive substrate and oxygen-containing moieties, which can be combined with NH_2_-VMSF to generate a stable and sensitive sensing interface. Carcinoembryonic antigen (CEA) is used as a model to examine the proposed sensing strategy. After the covalent modification of CEA-specific aptamer on the external surface of NH_2_-VMSF/p-SPCE with the help of cross-linking agent, access of electrochemical probes [Fe(CN)_6_^3−/4−^] to the underlying p-SPCE is controlled by the formation of the aptamer-CEA complex at the electrode surface and thereby results in the quantitative relationship between the electrochemical current signals of Fe(CN)_6_^3−/4−^ and the logarithm of CEA concentration. The proposed gated electrochemical aptasensing strategy based on the NH_2_-VMSF/p-SPCE enables the analysis of CEA in spiked human serum, providing a new avenue for the design of disposable and sensitive electrochemical aptasensors and expanding the analytical application of VMSF.

## 2 Materials and methods

### 2.1 Chemicals and materials

Carcinoembryonic antigen (CEA), alpha-fetoprotein (AFP), carbohydrate antigen 125 (CA 125), carbohydrate antigen 19–9 (CA 19–9), and fetal bovine serum were purchased from Beijing KeyGen Biotech Co., Ltd. (Beijing, China). Amino-modified CEA aptamer (5′-ATACAGCTTCAATT-NH_2_-3′) (Yan et al., 2023) was purchased from Sangon Biotechnology Co., Ltd (Shanghai, China). Prostate-specific antigen (PSA) was procured from Beijing Biodragon Immunotechnologies Co., Ltd. (Beijing, China). Tetraethyl orthosilicate (TEOS, 98%), cetyltrimethylammonium bromide (CTAB), bovine serum albumin (BSA), glutaraldehyde (GA), sodium dihydrogen phosphate dihydrate (NaH_2_PO_4_·2H_2_O), disodium hydrogen phosphate dodecahydrate (Na_2_HPO_4_·12H_2_O), sodium hydroxide (NaOH), potassium ferricyanide [K_3_Fe(CN)_6_], and potassium ferrocyanide [K_4_Fe(CN)_6_] were purchased from Aladdin Bio-Chem Technology Co., Ltd. (Shanghai, China). 3-Aminopropyltriethoxysilane (APTES) and potassium hydrogen phthalate (KHP) were purchased from Shanghai McLean Reagent Co., Ltd (Shanghai, China). Sulfuric acid, acetone, anhydrous ethanol (99.8%), and concentrated hydrochloric acid (HCl, 36%–38%) were obtained from Shuanglin Reagent Co., Ltd. (Hangzhou, China). Sodium nitrate (NaNO_3_) was ordered from Hangzhou Gaojing Fine Chemical Co., Ltd. (Hangzhou, China). Screen-printed carbon electrodes (SPCEs) were purchased from Metrohm (Bern, Switzerland).

In brief, SPCEs contain three integrated electrodes, namely, a working electrode (4 mm diameter), a counter electrode made up of conductive graphite paste, and an Ag reference electrode comprising of conductive silver paste. Phosphate-buffered saline (PBS, 0.01 M, pH = 7.4) was prepared using NaH_2_PO_4_·2H_2_O and Na_2_HPO_4_·12H_2_O. All the aqueous solutions used here were prepared using ultrapure water (18.2 MΩ cm) from Milli-Q Systems (Millipore Inc., Massachusetts, America). All chemical reagents were of analytical grade.

### 2.2 Characterization and instrumentation

The morphology and thickness of NH_2_-VMSF were characterized using transmission electron microscopy (TEM, model HT7700, Hitachi, Tokyo, Japan). To prepare TEM samples, the NH_2_-VMSF layer was carefully scraped off the electrode using a scalpel and dispersed in anhydrous ethanol with subsequent ultrasonic dispersion. Then, the resulting dispersion was drop-cast onto a copper grid. Before morphology characterization under 200 kV, the sample was dried naturally. All electrochemical experiments, including cyclic voltammetry (CV), electrochemical impedance spectroscopy (EIS), and differential pulse voltammetry (DPV), were conducted on an Autolab electrochemical workstation (model PGSTAT302N, Metrohm Autolab, Switzerland). The frequency range for EIS measurements was from 0.1 Hz to 100 kHz, with a perturbation amplitude of 5 mV.

### 2.3 Preparation of NH_2_-VMSF/p-SPCE

NH_2_-VMSF was grown on the surface of a p-SPCE electrode using the EASA method, as reported in the literature (Ma et al., 2022b). Bare SPCE was electrochemically polished by CV scanning in diluted H_2_SO_4_ (0.05 M) 10 times at a potential of 0.4 V–1.0 V. Then, the electrode was thoroughly washed with ultrapure water and dried with nitrogen. After that, the cleaned SPCE was subjected to electrochemical polarization. Specifically, a constant potential of +1.8 V was applied to SPCE for 300 s to perform anodic oxidation, followed by cathodic polarization in PBS (0.1 M, pH = 5) scanning from −1.3 V to +1.25 V. The resulting electrode is called p-SPCE.

To grow NH_2_-VMSF on the p-SPCE, a mixture of 20 mL ethanol, 20 mL NaNO_3_ solution (0.1 M, pH = 2.36), CTAB (1.585 g), and APTES (318 μL) was prepared. The pH of the solution was adjusted to 2.97 using HCl before TEOS (2,732 μL) was added. Then, the solution was vigorously stirred and reacted for 2.5 h to obtain the precursor solution. Subsequently, the p-SPCE electrode was put into the precursor solution and subjected to the constant current (current density: −0.74 mA/cm^2^, duration: 10 s) to grow NH_2_-VMSF on the p-SPCE surface. The resulting electrode was washed with ultrapure water, dried with nitrogen, and aged at 80°C overnight to obtain the p-SPCE modified with a hybrid film consisting of surfactant micelles (SMs) and NH_2_-VMSF, which was named SM@NH_2_-VMSF/p-SPCE. Finally, the SM@NH_2_-VMSF/p-SPCE electrode was immersed in an HCl-ethanol solution (0.1 M) and stirred for 5 min to remove SMs from silica nanochannels, yielding NH_2_-VMSF/p-SPCE with open nanochannels.

### 2.4 Fabrication of the BSA/Apt/GA/NH_2_-VMSF/p-SPCE aptasensor

NH_2_-VMSF/p-SPCE was used to construct an electrochemical aptasensor for CEA detection by using GA as a cross-linking agent, finally generating the amino groups on the outer surface of NH_2_-VMSF and further immobilizing CEA-specific aptamer (Apt). Specifically, to modify the GA only on the outer surface of NH_2_-VMSF, the amino groups on the outer surface were first derivatized with aldehyde groups before removing the SMs. A 1% GA solution was dropped onto the SM@NH_2_-VMSF/p-SPCE and incubated at 37°C in the dark for 20 min. After thorough washing, the electrode was soaked in a 0.1 M HCl-ethanol solution and stirred for 5 min to remove SMs. The resulting electrode was denoted as the GA/NH_2_-VMSF/p-SPCE. Next, a 100 mM Apt solution in 0.01 M PBS (pH 7.4) was dropped onto the GA/NH_2_-VMSF/p-SPCE carrying aldehyde moieties and incubated at 4°C for 90 min. The electrode was then washed with 0.01 M PBS (pH 7.4) to remove any unbound Apt to obtain Apt/GA/NH_2_-VMSF/p-SPCE. A 0.1 wt% solution of BSA in 0.01 M PBS (pH 7.4) was used to incubate the Apt/GA/NH_2_-VMSF/p-SPCE at 4°C for 30 min to block any nonspecific binding sites. The resulting aptasensor was denoted as BSA/Apt/GA/NH_2_-VMSF/p-SPCE.

### 2.5 Electrochemical detection of CEA

The BSA/Apt/GA/NH_2_-VMSF/p-SPCE aptasensor was incubated with different concentrations of CEA at 4°C for 60 min. The detection solution was a 0.1 M KCl solution containing 1.25 mM Fe(CN)_6_^3−/4−^. Electrochemical signals of Fe(CN)_6_^3−/4−^ were determined by the BSA/Apt/GA/NH_2_-VMSF/p-SPCE aptasensor before and after CEA binding using DPV. The standard addition and recovery methods were used to analyze the real samples of human serum from healthy adults without complicated pretreatments. The received human serum provided by healthy volunteers was diluted 50 times with PBS (0.01 M, pH = 7.4), and then various known amounts of CEA were added in turn to obtain real samples for detection.

## 3 Results and discussion

### 3.1 Fabrication of electrochemical aptasensors for sensitive detection of CEA based on NH_2_-VMSF/p-SPCE

**SCHEME 1:**
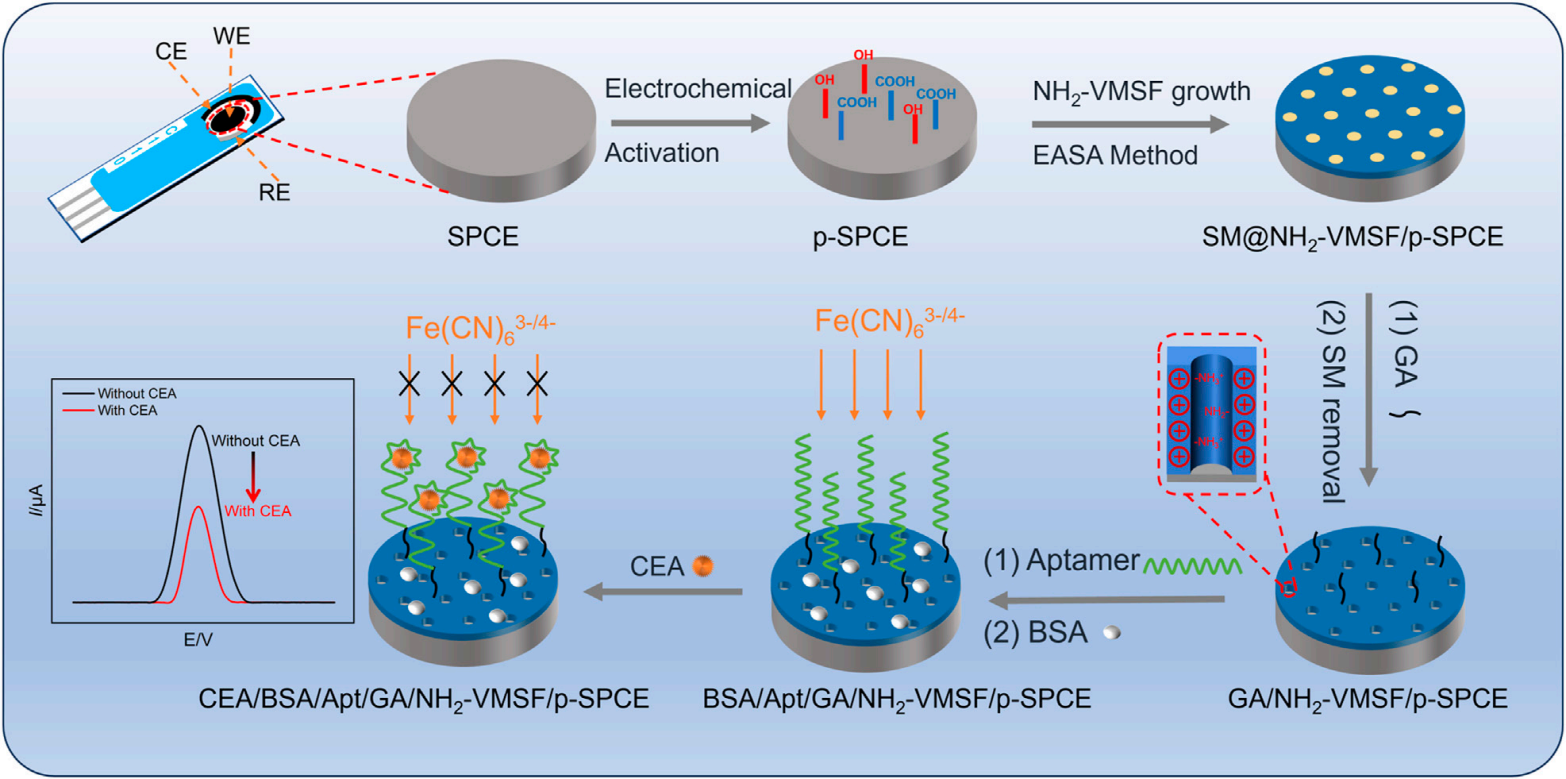
Schematic illustration for the preparation of the BSA/Apt/GA/NH_2_-VMSF/p-SPCE aptasensor and its electrochemical determination mechanism for CEA.

Combining the advantages of carbon electrodes and screen-printed electrodes, SPCE has the advantages of good chemical stability, a wide potential window, low cost, and easy mass production. As for the conductive property of SPCE, NH_2_-VMSF can be grown on SPCE using the EASA method but lacks stability. Therefore, prior to the fabrication of NH_2_-VMSF, bare SPCE undergoes an electrochemical pre-activation procedure (Scheme 1). As displayed, oxygen-containing functional groups appear at the p-SPCE and can form chemical bonds with silanol groups of NH_2_-VMSF to effectively increase the stability of NH_2_-VMSF on the p-SPCE. In addition, abundant defects presented in the pre-activation operation can boost the electrochemical activity of SPCE. NH_2_-VMSF directly obtained from the EASA method remains on the surfactant micelles (SMs) within the silica nanochannels and is employed to incubate with glutaraldehyde (GA) cross-linking agent to specifically generate aldehyde groups on the outer surface of NH_2_-VMSF. Note that SMs confined in the nanochannels of NH_2_-VMSF effectively prohibit the access and modification of GA on the inner nanochannels. After further covalent binding with CEA-specific aptamer (Apt) and blockage of nonspecific binding sites by bovine serum albumin (BSA), SMs are excluded using 0.1 M HCl-ethanol solution to obtain BSA/Apt/GA/NH_2_-VMSF/p-SPCE. The detection mechanism for CEA relies on the impeded diffusion of electrochemical probes [Fe(CN)_6_^3−/4−^] to the underlying p-SPCE upon the recognition of CEA. Based on the above principle, the quantitative relation between the decreased electrochemical current signals of Fe(CN)_6_^3−/4−^ and CEA concentration can be used to realize the detection of CEA.

### 3.2 Characterization of p-SPCE and NH_2_-VMSF/p-SPCE

**FIGURE 1:**
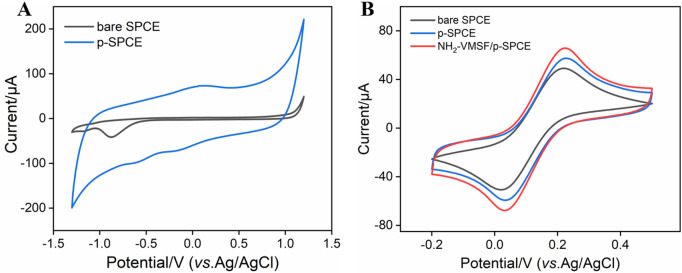
**(A)** CV curves of bare SPCE and p-SPCE in PBS (0.1 M, pH 5.0). **(B)** CV curves of bare SPCE and p-SPCE in 0.1 M PBS containing 1.25 mM Fe(CN)_6_^3−/4−^. The scan rate in **(A, B)** is 100 mV/s.

Considering the good electrocatalytic ability of p-SPCE, CV curves of bare SPCE and p-SPCE in 0.1 M PBS (pH 5.0) are compared. As seen in Figure 1A, compared with that of bare SPCE, apparent enhancement of changing current is observed at the p-SPCE, suggesting the increased electroactive area of p-SPCE after pre-activation treatment. Moreover, a pair of redox peaks near 0 V appeared at the p-SPCE and were assigned to the electrochemical reaction between hydroquinone and quinone. Figure 1B displays the CV responses of bare SPCE and p-SPCE to 1.25 mM Fe(CN)_6_^3−/4−^. Increased electrochemical redox currents are shown at the p-SPCE, confirming the improved electron transport property of p-SPCE and further allowing the design of highly sensitive electroanalytical sensors.

**FIGURE 2:**
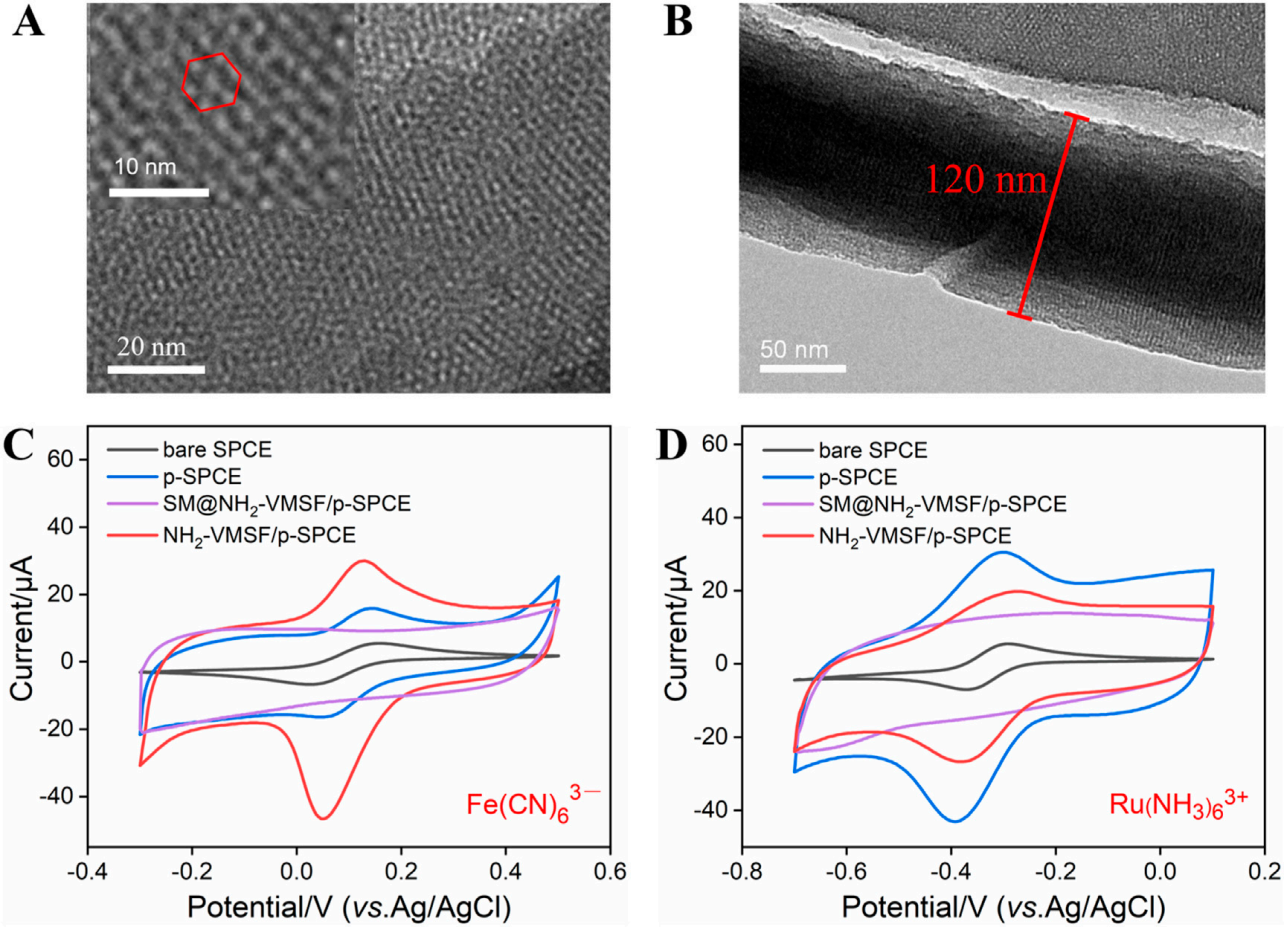
TEM characterization of NH_2_-VMSF: top-view **(A)** and cross-sectional view **(B)**. CV curves of bare SPCE, p-SPCE, SM@NH_2_-VMSF/p-SPCE, and NH_2_-VMSF/p-SPCE in a 50 mM KHP solution containing 0.5 mM K_3_ [Fe(CN)_6_] **(C)** and Ru(NH_3_)_6_Cl_3_ **(D)**. The scan rate in **(C, D)** is 50 mV/s.

NH_2_-VMSF scraped from p-SPCE was observed by TEM and the results are shown in Figures 2A, B. The top-view TEM image of NH_2_-VMSF exhibits many nanopores as bright spots with a pore diameter of 2–3 nm (Figure 2A). The cross-sectional TEM image of NH_2_-VMSF shows several nanochannels parallel to each other, with a uniform thickness of 120 nm (Figure 2B). Subsequently, different electrodes including bare SPCE, p-SPCE, SM@NH_2_-VMSF/p-SPCE, and NH_2_-VMSF/p-SPCE were employed to detect charged 0.5 mM Fe(CN)_6_^3−^ and Ru(NH_3_)_6_^3+^, and CV curves are shown in Figures 2C, D. As displayed, electrochemical pre-activation of SPCE can result in the increased charging currents and Faradic currents for these two probes, suggesting the increased electroactive area and accelerated electron transport ability of p-SPCE. Only charging currents for two probes are shown at the SM@NH_2_-VMSF/p-SPCE, indicating that the SMs inside the nanochannels can block the access of probes and the as-prepared NH_2_-VMSF on the p-SPCE is intact. NH_2_-VMSF has many open nanochannels, and its inner surface is rich in amino groups. Due to the protonation of amino groups, NH_2_-VMSF/p-SPCE exhibits a positively charged surface and electrostatic selectivity for the above two charged probes. By comparing the Faradic currents obtained at the p-SPCE, the Faradic currents for negatively charged Fe(CN)_6_^3−^ probe are significantly enhanced, and the signals for positively charged Ru(NH_3_)_6_^3+^ probe are decreased. This result indicates that the NH_2_-VMSF/p-SPCE with electrostatic effect has the ability to amplify Fe(CN)_6_^3−^ signals in the following quantitative analysis study. The above results confirm the successful fabrication of NH_2_-VMSF on the p-SPCE and are similar to the previous reports (Etienne et al., 2009).

### 3.3 Characterization for the stepwise construction of a BSA/Apt/GA/NH_2_-VMSF/p-SPCE aptasensor

**FIGURE 3:**
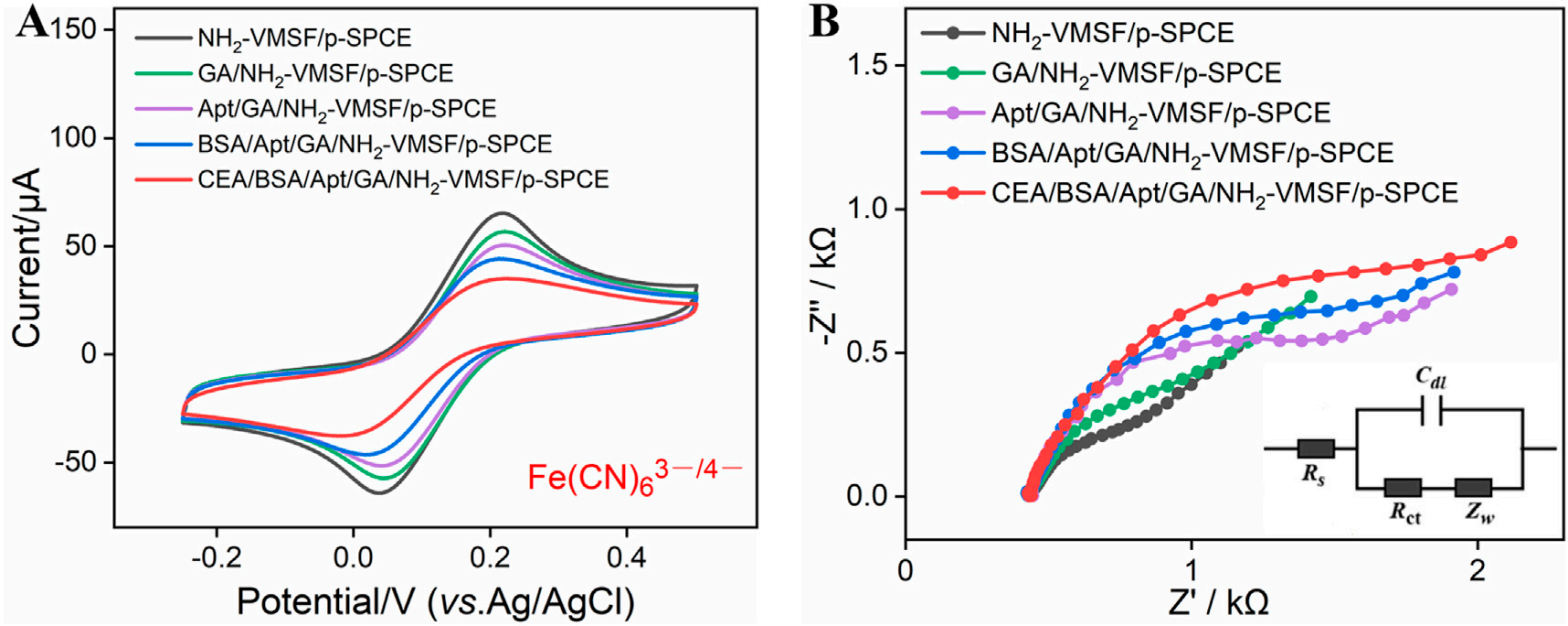
**(A)** CV curves of NH_2_-VMSF/p-SPCE, GA/NH_2_-VMSF/p-SPCE, Apt/GA/NH_2_-VMSF/p-SPCE, and BSA/Apt/GA/NH_2_-VMSF/p-SPCE before and after incubation of 100 pg/mL CEA in a 0.1 M KCl solution containing 1.25 mM Fe(CN)_6_^3−/4−^. The scan rate is 50 mV/s. **(B)** EIS curves obtained from the different electrodes in a 0.1 M KCl solution containing 2.5 mM Fe(CN)_6_^3−/4−^. The frequency range for EIS measurements was from 0.1 Hz to 100 kHz, with a perturbation amplitude of 5 mV.

NH_2_-VMSF/p-SPCE is a good electrode interface that can support the development of developing highly sensitive and disposable biosensors. To examine the ability of BSA/Apt/GA/NH_2_-VMSF/p-SPCE, CEA was used as a model and its corresponding CEA-specific aptamer as a recognition element was immobilized on the outer surface of NH_2_-VMSF/p-SPCE. The resulting BSA/Apt/GA/NH_2_-VMSF/p-SPCE aptasensor is achieved by stepwise modification of GA, Apt, and BSA (Scheme 1) and is characterized by CV and EIS techniques. As shown in Figure 3, consequent modification of GA, Apt, and BSA leads to decreased redox peak currents and enhanced semicircle diameter corresponding to the charge transfer resistance, which is due to the hindered diffusion of Fe(CN)_6_^3−/4−^ toward the electrode. When BSA/Apt/GA/NH_2_-VMSF/p-SPCE is incubated with 100 pg/mL CEA, further hindered diffusion for Fe(CN)_6_^3−/4−^ occurs and suggests the potential of our proposed aptasensor for quantitative determination of CEA.

### 3.4 Optimization of construction conditions of BSA/Apt/GA/NH_2_-VMSF/p-SPCE

**FIGURE 4:**
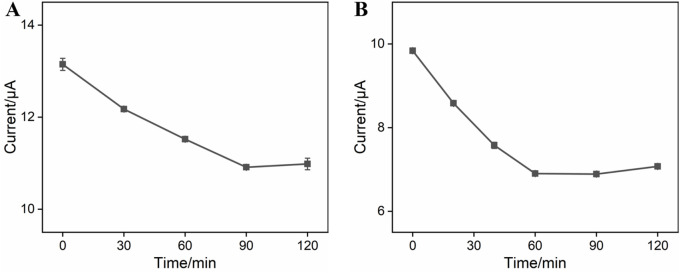
**(A)** Anodic peak currents obtained from DPV curves at GA/NH_2_-VMSF/p-SPCE after incubation with 0.1 μM CEA-specific aptamer under various times in a 0.1 M KCl solution containing 1.25 mM Fe(CN)_6_^3−/4−^. **(B)** Anodic peak currents obtained from DPV curves at the BSA/Apt/GA/NH_2_-VMSF/p-SPCE aptasensor after incubation with 10 pg/mL CEA under various times in a 0.1 M KCl solution containing 1.25 mM Fe(CN)_6_^3−/4−^. Error bars represent the standard deviation (SD) values of the results measured in three parallel experiments.

Experimental conditions for the construction of BSA/Apt/GA/NH_2_-VMSF/p-SPCE can influence the analytical performance of CEA. Therefore, incubation times for the CEA-specific aptamer and target CEA were studied, and the results are shown in Figure 4. As exhibited, anodic peak currents at the BSA/Apt/GA/NH_2_-VMSF/p-SPCE aptasensor decrease with the increasing incubation time at the beginning and subsequently tend to approach a plateau. The equilibrium times for the CEA-specific aptamer and target CEA of 90 min and 60 min, respectively, are used in the following study.

### 3.5 Electrochemical detection of CEA using the fabricated BSA/Apt/GA/NH_2_-VMSF/p-SPCE aptasensor

**FIGURE 5:**
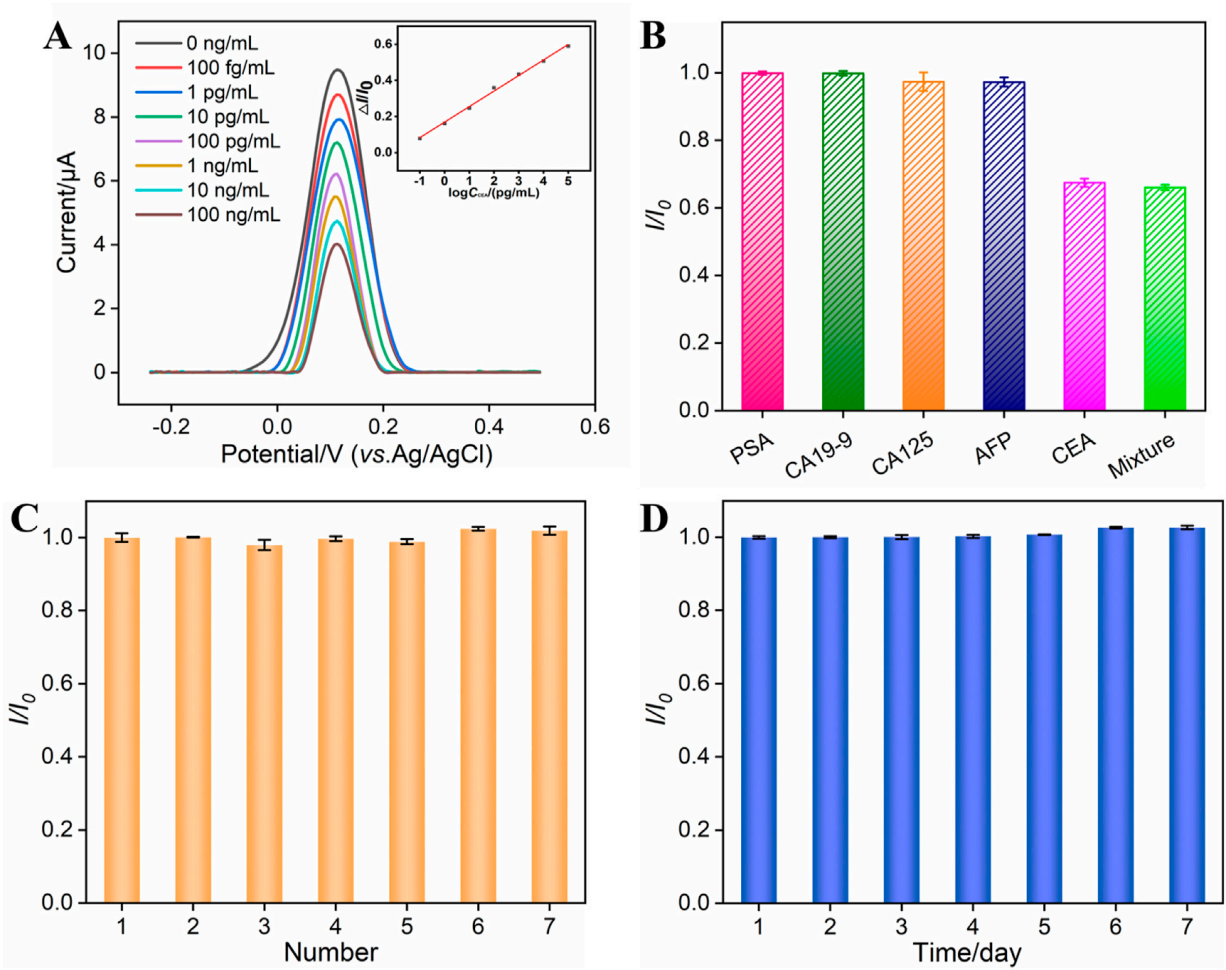
**(A)** DPV curves obtained from the BSA/Apt/GA/NH_2_-VMSF/p-SPCE aptasensor in a 0.1 M KCl solution containing 1.25 mM Fe(CN)_6_^3−/4−^ and various concentrations of CEA ranging from 100 fg/mL to 100 ng/mL. The inset is the corresponding calibration curve between △*I/I*_0_ and the logarithm of the CEA concentration. **(B)** Relative ratio (*I/I*_0_) of anodic peak currents at the BSA/Apt/GA/NH_2_-VMSF/p-SPCE aptasensor in a 0.1 M KCl solution containing 1.25 mM Fe(CN)_6_^3−/4−^ before (*I*_0_) and after (*I*) incubation with PSA (10 ng/mL), CA19-9 (10 U/mL), CA125 (1 mU/mL), AFP (10 ng/mL), CEA (0.1 ng/mL), or their mixture. **(C)** Reproducibility of the BSA/Apt/GA/NH_2_-VMSF/p-SPCE aptasensor. Peak current of the DPV anode obtained from seven parallel BSA/Apt/GA/NH_2_-VMSF/p-SPCE aptasensors in a 0.1 M KCl solution containing 1.25 mM Fe(CN)_6_^3−/4−^ after incubation with CEA. *I*_0_ and *I* are anodic peak currents obtained on the first aptasensor and other aptasensors. **(D)** Stability of the BSA/Apt/GA/NH_2_-VMSF/p-SPCE aptasensor. The prepared BSA/Apt/GA/NH_2_-VMSF/p-SPCE was placed at 4°C and used to detect 0.1 ng/mL CEA on different days. *I*_0_ and *I* are anodic peak currents obtained on the first day and other days. The error bars represent the SD of three measurements using three parallel electrodes.

The analytical performance of the BSA/Apt/GA/NH_2_-VMSF/p-SPCE sensor for CEA detection was evaluated by the DPV method. Several concentrations of CEA were incubated at the BSA/Apt/GA/NH_2_-VMSF/p-SPCE surface and tested in a 0.1 M KCl solution containing 1.25 mM Fe(CN)_6_^3−/4−^. As revealed in Figure 5A, anodic peak currents of Fe(CN)_6_^3−/4−^ gradually decline with an increase in CEA concentration. By plotting the anodic peak current ratio (△*I*/*I*_0_, where △*I* refers to *I* − *I*_0_, and *I* and *I*_0_ denote the anodic peak current in the presence and absence of CEA, respectively.) against the logarithm of the CEA concentration, a wide linear range of 100 fg/mL to 100 ng/mL is obtained. The corresponding linear regression equation is △*I*/*I*_0_ = 0.086 log *C*_CEA_ (pg/mL) + 0.1674 (*R* ^2^ = 0.997). The smallest discernible DPV signal is obtained by subtracting the standard deviation from the average anodic peak current of three-time blank signals, which is named *I*, and is substituted into the above linear regression equation to calculate the limit of detection (LOD). The LOD for CEA is estimated to be 24 fg/mL, which is lower than those of electrochemical sensors shown in Table 1 and demonstrates an excellent disposable and sensitive aptasensor.

**TABLE 1:** Analytical performance of our BSA/Apt/GA/NH_2_-VMSF/p-SPCE aptasensor and other electrochemical sensors for CEA detection.

Material	Method	Liner range (pg/mL)	LOD (pg/mL)	Ref.
dsDNA/MXC-Fe_3_O_4_-Ru/MGE	DPV	1–10^6^	0.62	Yang et al. (2022)
GNRs-aptamer/CEA/BSA/anti-CEA-GO/GCE	SWV	0.1–10^4^	0.05	Si et al. (2017)
hybrid DNA/CEA-H1/BSA/MCH/H2/AuE	DPV	10–10^5^	0.84	Niu et al. (2022)
pβ-pep/PANI/GCE	DPV	10^–3^−10^3^	3.3 × 10^−4^	Zhao et al. (2023)
Apt/MCH/cpDNA_2_/AuE	DPV	2 × 10^3^–4.5 × 10^4^	240	Zhai et al. (2021)
HRP@ConA/CEA/MCH-Apt/AuE	DPV	5 × 10^3^–4 × 10^4^	3.4 × 10^3^	Wang et al. (2018)
BSA/Apt/GA/NH_2_-VMSF/p-SPCE	DPV	0.1–10^5^	0.024	This work

dsDNA, double-stranded DNA; cpDNA, chloroplast DNA; MXC, carboxyl functionalized 2D nanomaterial MXene; MGE, magnetic gold electrode; GNRs, gold nanorods; GO, graphene oxide; GCE, glassy carbon electrode; H1, hairpin probe 1; MCH, 6-mercapto-1-hexanol; H2, hairpin probe 2; AuE, Au electrode; pβ-pep, peptide containing unnatural β-homoserine; PANI, polymer polyaniline; cpDNA2, capture DNA; HRP, horseradish peroxidase; ConA, concanavalin A.

The selectivity, reproducibility, and stability of our fabricated BSA/Apt/GA/NH_2_-VMSF/p-SPCE sensor were also verified, and the data are shown in Figures 5B–D. As presented in Figure 5B, PSA, CA19-9, CA125, and AFP were tested by a BSA/Apt/GA/NH_2_-VMSF/p-SPCE sensor in a 0.1 M KCl solution containing 1.25 mM Fe(CN)_6_^3−/4−^, respectively. The anodic peak current variations were compared with those of CEA and a mixture consisting of CEA and these four potential interfering species. The results indicate that the magnitude of anodic peak currents obtained at the BSA/Apt/GA/NH_2_-VMSF/p-SPCE remains unchanged in the presence of these four interfering species, implying that the proposed aptasensor enables the selective detection of CEA. Seven parallel BSA/Apt/GA/NH_2_-VMSF/p-SPCE aptasensors were used to detect 0.1 ng/mL CEA, displaying comparable electrochemical current variation and showing a good reproducibility of the as-prepared aptasensor (Figure 5C). The prepared BSA/Apt/GA/NH_2_-VMSF/p-SPCE without any solution was placed at 4°C and used to detect 0.1 ng/mL CEA on different days. Figure 5D shows that the BSA/Apt/GA/NH_2_-VMSF/p-SPCE aptasensor had excellent stability for the detection of 0.1 ng/mL CEA within 7 days.

### 3.6 Detection of CEA in human serum samples

The fabricated BSA/Apt/GA/NH_2_-VMSF/p-SPCE aptasensor was applied to detect CEA in human serum samples. After simple dilution treatment and the addition of a series of CEA samples with known concentrations, artificial human serum samples were obtained and measured by our BSA/Apt/GA/NH_2_-VMSF/p-SPCE aptasensor. As shown in Table 2, the detected concentrations (found) by BSA/Apt/GA/NH_2_-VMSF/p-SPCE aptasensor are comparable to those known spiked concentrations (added), exhibiting satisfactory recoveries and relative standard deviation (RSD) values.

**TABLE 2:** Determination of CEA in human serum samples.

Sample	Added (ng/mL)	Found (ng/mL)	Recovery (%)	RSD (%, n = 3)
Serum^a^	0.00100	0.00107	107	2.1
	0.100	0.0962	96.2	2.7
	10.0	9.96	99.6	1.1

^a^ Human serum samples are diluted by a factor of 50 using PBS (0.01 M, pH 7.4). The diluted concentrations of CEA are shown in the table.

## 4 Conclusion

In summary, NH_2_-VMSF/p-SPCE, combining a tailored rigid skeleton and an electroactive sensing substrate, was used for the design of disposable gate electrochemical aptasnsor. The electrochemical polarization procedure of SPCE can generate plentiful oxygen-containing groups to promote the stability of NH_2_-VMSF on p-SPCE and improve the catalytic ability for enhanced electroanalytical performance. The CEA-specific aptamer anchored on the external surface of NH_2_-VMSF/p-SPCE acts as the gatekeeper and then specifically recognizes the target CEA, resulting in the impeded ingress of Fe(CN)_6_^3−/4−^ and thereby enabling the quantitative analysis of CEA. A wide detection range (100 fg/mL to 100 ng/mL) and a low limit of detection (24 fg/mL) were demonstrated by this gated electrochemical aptasensor based on the NH_2_-VMSF/p-SPCE. Note that such an aptasensor can be used at least three times in the same sample before being disposed of. Moreover, analysis of CEA in spiked human serum samples is satisfied, which extends the biological applications of disposable NH_2_-VMSF/p-SPCE and allows the detection of various analytes by modulating the biological recognition species.

## Data Availability

The raw data supporting the conclusions of this article will be made available by the authors, without undue reservation.
